# Medicinal Herbs: Promising Immunomodulators for the Treatment of Infectious Diseases

**DOI:** 10.3390/molecules28248045

**Published:** 2023-12-12

**Authors:** Hamad H. Alanazi, Abdelbaset Mohamed Elasbali, Maged K. Alanazi, Eman Fawzy El Azab

**Affiliations:** Department of Clinical Laboratory Science, College of Applied Medical Sciences-Qurayyat, Jouf University, Al-Qurayyat 77455, Saudi Arabia; aeelasbali@ju.edu.sa (A.M.E.); efelazab@ju.edu.sa (E.F.E.A.)

**Keywords:** immunomodulators, immune response, infectious diseases, natural compounds, virus, bacteria, cytokines, cancer

## Abstract

Humans are constantly at high risk of emerging pandemics caused by viral and bacterial infections. The emergence of new pandemics is mainly caused by evolved viruses and bacteria that are highly resistant to existing medications. The rapid evolution of infectious agents demands the urgent investigation of new therapeutic strategies to prevent and treat these infections at an early stage. One of these therapeutic strategies includes the use of medicinal herbs for their antibacterial and antiviral properties. The use of herbal medicines as remedies is very ancient and has been employed for centuries. Many studies have confirmed the antimicrobial activities of herbs against various pathogens in vitro and in vivo. The therapeutic effect of medicinal herbs is mainly attributed to the natural bioactive molecules present in these plants such as alkaloids, flavonoids, and terpenoids. Different mechanisms have been proposed for how medicinal herbs enhance the immune system and combat pathogens. Such mechanisms include the disruption of bacterial cell membranes, suppression of protein synthesis, and limitation of pathogen replication through the inhibition of nucleic acid synthesis. Medicinal herbs have been shown to treat a number of infectious diseases by modulating the immune system’s components. For instance, many medicinal herbs alleviate inflammation by reducing pro-inflammatory cytokines (e.g., tumor necrosis factor-alpha (TNF-α), interleukin-1, IL-6) while promoting the production of anti-inflammatory cytokines (e.g., IL-10). Medicinal herbs also play a role in defense against viral and intracellular infections by enhancing the proliferation and functions of natural killer cells, T-helper-1 cells, and macrophages. In this review, we will explore the use of the most common herbs in preventing and treating infectious and non-infectious diseases. Using current and recently published studies, we focus on the immunomodulatory and therapeutic effects induced by medicinal herbs to enhance immune responses during diseases.

## 1. Introduction

### 1.1. Immunomodulatory Effects of Innate and Adaptive Immune Systems

The immune system is the main barrier against harmful invaders like viruses, bacteria, and fungi. It comprises many organs, tissues, proteins, and cells including bone marrow, thymus, spleen, complement proteins, and leukocytes. Each of these components has a specific role in forming immunity against foreign invaders. The bone marrow is where almost all blood cells originate (e.g., dendritic cells, B-cells), and the thymus is the place for the maturation of T-cells. The immune system has two parts: innate and adaptive. The innate immune system is the first line of defense against foreign invaders. It consists of physical, biological, and chemical barriers that ensure the elimination of pathogens. Innate immune cells such as macrophages, dendritic cells, and natural killer cells (NK) can recognize and respond to foreign pathogens through pattern recognition receptors (PRRs) found in the endosome or on the cell membrane [1]. The recognition of microbial particles by PRRs leads to their activation and thus the release of cytokines and chemokines which promote inflammation and recruit other immune cells (such as neutrophils) to the site of the infection [1]. Moreover, innate immune cells can phagocytose and eliminate pathogens directly. For instance, many neutrophils phagocytose bacteria during bacterial infections and destroy them through enzymatic activities by forming reactive oxygen species (ROS), such as superoxide radicals, and hydrogen peroxide [2]. When the innate immune system fails to clear the pathogen, the adaptive immune system steps in to stop the infection. At this stage, the innate immune system regulates adaptive immune responses by presenting antigens to T-cells through dendritic cells and macrophages and producing cytokines that enhance T- and B-cell differentiation and proliferation [3].

In the case of intracellular infections caused by viruses or bacteria, natural killer cells and phagocytes such as macrophages destroy infected cells and engulf the pathogen, respectively. Infected cells then produce cytokines such as interferons which interfere with pathogen replication inside the host cells and recruit other immune cells to the site of infection [4]. Importantly, when natural killer cells and phagocytes fail to seize the intracellular infections, these cells present small fragments of the foreign antigen on the cell surface through a molecule called major histocompatibility complex-I (MHC-I). This way, the adaptive immune cells (i.e., CD8 or cytotoxic T-cells) are now alerted to an intracellular invader. Once cytotoxic T-cells are fully activated, they kill the infected cells with cytotoxic proteins called granzymes and perforins [5].

During extracellular infections by agents like bacteria or parasites, complement proteins and phagocytes including neutrophils destroy the pathogen [1]. However, when innate immunity fails, antigen-presenting cells such as dendritic cells phagocytose the pathogen and migrate to secondary lymphoid organs such as the lymph nodes. There, they present antigenic peptides through MHC-II to T-helper cells which aid B-cells in the production of antibodies [6]. Antibodies are highly efficient in combating extracellular agents through neutralization, blocking, and facilitating the process of phagocytosis [7].

In some cases, the immune system induces weak or insufficient immune responses towards pathogens and thus requires immunomodulators. Immunomodulators have been shown to be very therapeutically useful in the context of numerous diseases such as autoimmune diseases, cancers, and microbial infections [8,9]. In addition, immunomodulators have also been tested in reducing or suppressing overactive immune responses [10]. There are different types of immunomodulators that work as immunosuppressants, such as cyclosporine and tacrolimus which act by inhibiting T-cell activation in autoimmune diseases like rheumatoid arthritis and lupus [11,12]. However, many immunomodulators called immunostimulants act by enhancing immune responses during microbial infections or tumors. These include interferons, which enhance the functions of NK cells, and interleukins, which promote the proliferation and differentiation of T- and B-cells during disease [13].

**Figure 1:**
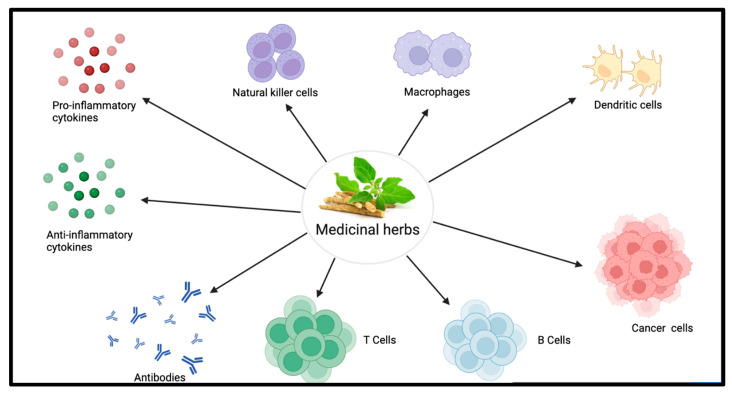
Immune components are affected by medicinal herbs. Medicinal herbs modulate the immune system in a number of ways like suppressing inflammatory cytokines and increasing anti-inflammatory cytokines or certain classes of antibodies.

Several studies have shown that phytochemicals or plant-derived compounds have beneficial immunomodulatory effects (Table 1). We have specifically selected these medicinal herbs because of their well-documented therapeutic properties. People have been using herbs as treatments for thousands of years [14]. In experimental studies, some of these herbs have been proven to be effective in treating disease in animal models and clinical settings (Figure 1). However, the mechanisms of how these natural compounds work are not clear. Several important bioactive compounds are commonly found in herbs that produce similar effects. In this review, we focus on defining the main bioactive components in common phytochemicals and which of these components are shared with other phytochemicals, as well as how these components modulate immune responses therapeutically.

### 1.2. Examples of Therapeutically Important Medicinal Herbs

#### 1.2.1. Ashwagandha (*Withania somnifera*)

Ashwagandha (*Withania somnifera*) is a medicinal herb that has been used for ages to treat various medical conditions like anxiety, stress, diabetes, epilepsy, arthritis, and many inflammatory diseases [45,46]. *W. somnifera*’s bioactive components such as alkaloids and withanolides induce several immunomodulatory effects that enhance the immune system. It increases the proliferation and activity of T-cells, natural killer cells, and macrophages [15]. *W. somnifera* also induces anti-inflammatory effects and reduces pro-inflammatory effects through modulating cytokines such as IL-4 [15]. Studies testing the immunomodulatory role of *W. somnifera* in vivo resulted in very promising therapeutic effects. In a study using APP/PS1 transgenic mice as a model for Alzheimer’s disease, oral administration of *W. somnifera* semi-purified extract for 30 days reversed the phenotypes of Alzheimer’s disease such as behavioral impairments, plaque development, and the buildup of beta-amyloid peptides (Aβ) and oligomers in the brains of middle-aged and elderly APP/PS1 transgenic mice [47]. In cancer studies, *W. somnifera* was shown to be a highly effective treatment in vitro and in vivo. The study suggested that *W. somnifera* induces its antitumor activities by generating ROS and inducing apoptosis in new cancer cells while tolerating normal cells [48]. Moreover, *W. somnifera* was shown to promote chemotherapy through the enhancement of mitochondrial dysfunction in cancerous cells only [49]. *W. somnifera* was also tested for its therapeutic value in metabolic diseases like diabetes. It was shown that *W. somnifera* can restore urine sugar, blood glucose, and glycosylated hemoglobin (HbA1C) in rat diabetic models (alloxan-induced diabetes mellitus (DM) rats) [50]. Additionally, *W. somnifera* also plays a therapeutic role in inflammation and chronic diseases like arthritis. A study by khan et al. showed that the oral administration of *W. somnifera* root at a dose of 300 mg/kg reduces the inflammatory cytokines (e.g., TNF-α, IL-1β, IL-6) which are usually high in arthritic rats [51]. The study also showed that *W. somnifera* root induces an increase in anti-inflammatory cytokines (i.e., IL-10) and the suppression of NF-κB, which is an important transcription factor for inflammation [51]. These findings suggest a great therapeutic role for *W. somnifera* in the treatment of inflammatory diseases like arthritis.

#### 1.2.2. Astragalus (*Astragalus membranaceus*)

Astragalus (*Astragalus membranaceus*) is a traditional Chinese medicinal herb that has been used for centuries to treat a variety of diseases. *A. membranaceous* has many anti-inflammatory, antioxidant, and immunoregulatory effects [52]. The main components of *A. membranaceous* are polysaccharides, flavonoids, and saponins. Additionally, astragalus has been used for its antitumor activities as in vivo studies demonstrated elevated levels of natural killer (NK) cells and NK-derived interferon-γ (IFN-γ) after the administration of astragalus [18]. The *A. membranaceous* component polysaccharide increases T-helper (Th1) cell numbers [53]. It seems that astragalus polysaccharide (APS) is the main active component in *A. membranaceous*. Many studies investigated the role of APS in several chronic diseases. It was shown that APS has a protective role in diseases like diabetes, renal injuries, and myocardial dysfunction [54,55,56]. In addition to APS, the *A. membranaceous* component astragaloside IV has also been shown to modulate immune responses by increasing both cellular and humoral immunity. Astragaloside IV increases T- and B-cell proliferation, antibody production, and anti-inflammatory cytokines such as transforming growth factor-β (TGF-β), but reduces pro-inflammatory cytokines such as TNF-α and IL-1 [57,58].

In vivo studies testing the therapeutic immunomodulatory role of *A. membranaceous* have shown promising results. The *A. membranaceous* component astragalus polysaccharides (APS) improved immunity and reversed immunosuppression and microbial dysbiosis [59]. Moreover, the *A. membranaceous* chemical component astragaloside IV (as-IV) was shown to attenuate myocardial ischemia–reperfusion injury in rats by regulating the PI3K/AKT/GSK-3β signaling pathways, thereby improving cardiovascular health [60]. The therapeutic effects of *A. membranaceous* extended to chronic diseases like diabetic nephropathy and kidney dysfunction [61,62]. It was noted that blood pressure and urinary albumin were drastically reduced in chronic kidney disease (CKD) rat models treated with *A. membranaceous* compared to CKD rats that were untreated [62]. Moreover, *A. membranaceous* extract was shown to suppress the proliferation of breast cancer cells via the PI3K/AKT/mTOR signaling pathway, suggesting an alternative antitumor strategy [63]. The *A. membranaceous*-extracted polysaccharide called PG2 was reported to suppress the expression of programmed cell death protein ligand 1 (PD-L1), which contributes to cancer immunotherapy; thereby, this could be used as an effective approach when combined with other treatments [64].

#### 1.2.3. Echinacea (*Echinacea purpurea*)

*Echinacea purpurea* (*E. purpurea*) is a medicinal herb that has been used for centuries to treat a number of diseases like cold and flu. *E. purpurea* has multiple therapeutic properties including immunomodulatory, anti-inflammatory, antibacterial, and antiviral effects. The beneficial effects of *E. purpurea* are attributed to its main bioactive components including alkamides, caffeic acid derivatives, and polysaccharides [65]. Both cellular and humoral immunity were enhanced by *E. purpurea* [66]. Studies also showed that T- and B-lymphocytes as well as NK cell proliferation and activity were increased following *E. purpurea* extract treatment [67]. In vivo experiments have suggested that *E. purpurea* extract has several immunomodulatory effects [67]. The oral administration of *E. purpurea* extract in a mouse model was shown to elevate MHC II and increase several immune components such as CD4+ T-cells, Th1 cytokines, and immunoglobulin levels [67]. In addition to the role of *E. purpurea* in immunomodulation, it was shown that *E. purpurea* extract can reduce inflammation markers in vitro and in vivo through signaling pathways involving ERK1/2, p38, STAT3, and cyclooxygenase-2 (COX-2) [68,69]. Moreover, the therapeutic activity of *E. purpurea* extract was tested for wound healing. In a model wounds of HaCaT cells, it was reported that *E. purpurea* extract promoted significant wound healing, demonstrated by the migration of keratinocytes and fibroblasts to the wound (scratch in cell monolayer) [70]. *E. purpurea* extract is also efficient in treating chronic disorders such as diabetes mellitus (DM) [71]. Additionally, it was reported in this study that the administration of *E. purpurea* extract for about 4 weeks significantly improved hyperglycemia and insulin resistance in a diabetic rat model [71]. Furthermore, *E. purpurea* extract improved other medical conditions usually linked with diabetes and oxidative stress leading to infertility. It was shown that *E. purpurea* extract preserved sperm motility, sperm morphology, and mitochondrial membrane potential [71].

#### 1.2.4. Garlic (*Allium sativum*)

Garlic (*Allium sativum*) is a very common natural product used for culinary and healing purposes. *A. sativum* has been shown to treat bacterial, fungal, and viral infections [72]. Many of the therapeutic effects of *A. sativum* are attributed to a substance called “Allicin” which is the major natural sulfur compound released when garlic is crushed [72]. *A. sativum* has been used as a therapeutic in a number of chronic medical conditions. In cardiovascular diseases, it reduces cholesterol levels and high blood pressure, and it has been shown to have many immunomodulatory effects [73]. As an immunomodulator, *A. sativum* was shown to inhibit inflammation by suppressing IL-6, MCP-1, TNF-*α*, and NF-*κ*B activity [23]. *A. sativum* was shown to have antiviral activity; for instance, *A. sativum* defends against human immunodeficiency virus (HIV) by inhibiting virus adhesion to host cells [74]. It also possesses antiviral activities against other viruses such as SARS-CoV-2, herpes simplex virus (HSV)-1 and -2, and others [74].

*Allium sativum* has been shown to have a huge impact on diabetic patients by improving insulin sensitivity and regulating blood glucose [75]. It has robust inhibitory activities against both Gram-positive and Gram-negative bacteria [76]. As a result of using antibiotics, many bacterial strains develop resistance to different antibiotics, such as the case of certain resistant strains of Clostridium perfringens and Escherichia coli, which cannot be killed by penicillin but are sensitive to garlic [77]. These data support the presence of the immunomodulatory effects of garlic on infections. In addition to the role of *A. sativum* in microbial diseases, some studies also showed that its therapeutic effects extend to different types of cancer. It was shown that *A. sativum* seizes the proliferation of cancer cells and stops the metastasis of the tumors by promoting the apoptosis of cancer cells [78]. Moreover, allcian, which is the main bioactive compound in *A. sativum*, was shown to suppress angiogenesis, thus promoting tumor regression [79].

#### 1.2.5. Ginger (*Zingiber officinale*)

Ginger (*Zingiber officinale*) has been used to treat diseases or alleviate inflammation for many years. The main bioactive components in *Z. officinale* are gingerols and shogaols, which promote anti-inflammatory and antioxidant immunomodulatory effects [80]. *Z. officinale* reduces the symptoms of inflammation and certain diseases through a number of immunomodulatory effects such as negatively modulating pro-inflammatory cytokines (e.g., TNF-α, IL-1β, and IL-6) while increasing anti-inflammatory cytokines such as IL-10 [30,81]. *Z. officinale* also has antiviral and antibacterial properties that can help treat various microbial infections [82]. In the case of viral infections where Th1 cells and interferons play an important role, ginger increases the proliferation and activation of Th1 cells and the production of IFN-γ cytokines, which are important for defense against infections caused by intracellular pathogens [83]. The effects of *Z. officinale* extend to B-cells. *Zingiber officinale* is known to promote the production of antibodies such as IgG and IgA by B-cells [84].

*Zingiber officinale* has been used in the treatment of various medical conditions like osteoarthritis, as well as joint and muscle discomfort, and neurological conditions [85]. It has also been used as a treatment for toothaches, asthma, diabetes, and constipation [85]. In diabetic patients, the oral administration of *Z. officinale* modulates various biological markers such as fasting blood sugar levels, hemoglobin A1c levels, apolipoprotein B, and apolipoprotein A-I [86]. In cancer studies, it was shown that *Z. officinale* stimulates apoptosis in multiple cancer types including ovarian, colon, breast, cervical, and prostate cancer [87]. Overall, ginger promotes several therapeutic benefits in a number of diseases and cancer forms; however, clinical trials to test its efficacy in treating and preventing cancer are still ongoing.

#### 1.2.6. Ginseng (*Panax ginseng*)

Ginseng (*Panax ginseng*) is a very common herb used for the treatment of numerous diseases such as high blood pressure, cancer, cardiovascular diseases, and hepatitis C [88,89]. *P. ginseng* has anti-inflammatory, antioxidant, and antitumor properties [90]. Similar to *Z. officinale*, *P. ginseng* promotes several immunomodulatory effects through immune system components. It was shown to enhance the production of anti-inflammatory cytokines (e.g., IL-10) and suppress the production of pro-inflammatory cytokines like TNF-α, IL-1β, IL-1, and IL-6 [91,92]. *P. ginseng* was also shown to enhance the proliferation and activation of lymphocytes including T-cells, B-cells, and NK cells, and the production of different subsets of immunoglobulins [93,94].

#### 1.2.7. Licorice (*Glycyrrhiza glabra*)

Licorice (*Glycyrrhiza glabra*) has been used as a traditional medicine to treat a wide range of diseases, including respiratory disorders, gastrointestinal disorders, skin conditions, and inflammatory diseases [95,96,97]. The main bioactive compounds in *G. glabra* are flavonoids, triterpenoids, and saponins which are believed to be responsible for the anti-inflammatory, antioxidant, antiviral, and immunomodulatory effects of *G. glabra* [98,99]. *G. glabra* was shown to have anti-inflammatory features. It suppresses pro-inflammatory cytokines, such as interleukin-6 (IL-6) and tumor necrosis factor-alpha (TNF-α) [98]. *G. glabra* was also shown to enhance the immune system against viral infections [100]. It increases T-cell activation and promotes T-regulatory cells (Treg). NK cell activity was enhanced by *G. glabra*, which plays a vital role in defense against viruses and cancerous cells [101,102]. In addition, *G. glabra* was shown to enhance humoral immunity through increasing B-cell activation and proliferation [103].

#### 1.2.8. Shatavari (*Asparagus racemosus*)

Shatavari (*Asparagus racemosus*) is also a traditional herb that has been used for ages to treat several diseases due to its role as an immunomodulator [104,105]. As in many herbs, the active components responsible for the immune responses in *G. frondosa* are saponins, flavonoids, and polysaccharides [106]. *A. racemosus* has been shown to enhance the activity of various immune cells like macrophages, neutrophils, and natural killer (NK) cells, thus contributing to defense against bacterial and viral infections [107]. *A. racemosus* was shown to inhibit pro-inflammatory cytokines, such as tumor necrosis factor-alpha (TNF-α), interleukin 1β, interleukin-6 (IL-6), and nitric oxide, while upregulating Th1 and Th2 cytokines (IL-2 and IL-4), thereby balancing the cytokine-involved immune response [108,109].

#### 1.2.9. Tulsi (*Ocimum sanctum*)

Tulsi (*Ocimum sanctum*) is very commonly used in India as an herbal plant to treat various medical conditions. It has been shown to alleviate inflammatory responses and boost the immune system in a number of ways. *O. sanctum* has been shown to increase the levels of IL-4, IFN-γ, and NK and T-cells [43]. It has been suggested that *O. sanctum* induces both bacterial and viral activities against a number of pathogens [110,111]. The active components responsible for most antimicrobial effects of Tulsi are terpenes, phenolics, phenolic acids, and flavonoids [112].

In addition to these medicinal herbs, there are several other herbs listed in (Table 2) that contribute to antimicrobial defense. The active herbal components with immunomodulatory effects are described in (Table 2).

## 2. Discussion

Natural compounds and molecules, derived from plants, represent an integral part of the development of therapeutic agents. The therapeutic role of bioactive compounds has been evaluated in a number of microbial diseases. It was shown that these compounds enhance the immune system in a number of ways, such as balancing pro- and anti-inflammatory cytokines (Figures S1–S10). Although there are studies showing the robust therapeutic effects of natural products, there are few clinical trials that practically test the benefits of natural products in serious diseases. One obstacle preventing the inclusion of natural products in many clinical trials is the absence of comprehensive data regarding both their safety and underlying mechanisms of action. In this review, we attempted to explore the recent therapeutic effects of natural products in a number of diseases. Certain products such as flavonoids play an immunomodulatory role in several medical conditions, suggesting their potential as candidates for treating infections.

## Figures and Tables

**Table 1:** Overview of different immune responses induced by medicinal herbs.

		Immunomodulatory Effects						
Medicinal Herbs	Bioactive Component	T-Cells	B-Cells	NK Cells	Antibodies	Pro-Inflammatory Cytokines	Anti-Inflammatory Cytokines	References
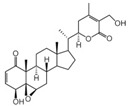 Ashwagandha (*Withania somnifera*)	withanolides	+	+	+	IgA, IgM, IgG, IgG2, IgG3 and IgG4	-	+	[15,16]
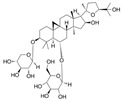 Astragalus (*Astragalus membranaceus*)	polysaccharides, saponins, flavonoids, astragalosides, lipopolysaccharides	+	+	+	IgG, IgA, IgM	-	+	[17,18,19,20]
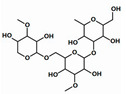 Echinacea (*Echinacea purpurea*)	alkamides, phenolic compounds	+	+	+	+	-	+	[21,22]
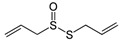 Garlic (*Allium sativum*)	allicin, diallyl sulfide (DAS), Z-ajoene	+	+	+	IgG, IgA	-	+	[23,24,25,26,27,28]
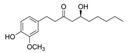 Ginger (*Zingiber officinale*)	gingerol, polyphenols, shogaols, paradols, zingerone	+	+	+	Total IgG, IgG1	-	+	[29,30,31,32,33]
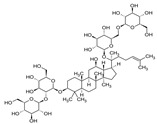 Ginseng (*Panax ginseng*)	ginsenosides, polysaccharides	+	+	+	IgM, IgG1, IgG2a, IgG2b	-	+	[34,35,36]
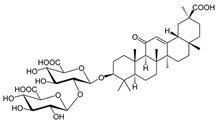 Licorice (*Glycyrrhiza glabra*)	triterpenoids, saponin, flavonoids, sterols	+	+	+	IgG, IgM	-	+	[37,38,39,40,41]
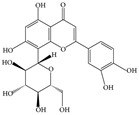 Tulsi (*Ocimum sanctum*)	saponins, flavonoids, terpenoids, linoleic acid	+	-	+	+	+	+	[42,43,44]

(+) indicates an increase in the specified immunomodulatory component, (-) indicates a decrease in the specified immunomodulatory component.

**Table 2:** List of herbal active components responsible for induction of immune responses.

Medicinal Plants	Active Components	References
1. Ashwagandha (*Withania somnifera*)	Alkaloids, flavonoids, steroids, tannins, withanolides, and glycosides	[46,113,114]
2. Astragalus (*Astragalus membranaceus*)	Astragalus polysaccharide (APS), alkaloids, saponins, and flavonoids	[115,116]
3. Echinacea (*Echinacea purpurea*)	Alkamides, polysaccharides, caffeic acid derivatives, and cichoric acid	[65,117]
4. Garlic (*Allium sativum*)	Allicin, diallyl sulfide (DAS), diallyl disulfide (DADS), diallyl trisulfide (DATS), alliin, S-allyl-cysteine, and Z-ajoene	[118]
5. Ginger (*Zingiber officinale*)	polyphenols (Gingerols), shogaols, terpenes, polysaccharides, organic acids, and paradols	[119]
6. Ginseng (*Panax ginseng*)	Ginsenosides, gintonins, and polysaccharides	[120]
7. Licorice (*Glycyrrhiza glabra*)	Triterpenoids, saponin (glycyrrhetinic acid), flavonoids and sterols	[121]
8. Shatavari (*Asparagus racemosus*)	Saponins, polysaccharides, flavonoids, polyphenols, and alkaloids	[122]
9. Tulsi (*Ocimum sanctum*)	alkaloids, saponins, flavonoids, terpenoids, linoleic acid, tannins, glycosides, carbohydrates, and proteins	[123]
10. Turmeric (*Curcuma longa*)	Curcuminoids, flavonoids, phenolic acid, amino acids, and sesquiterpenes	[124]
11. Andrographis (*Andrographis paniculata*)	Andrographolide, flavonoids, neoandrographolide, andrographine, and panicolines	[125]
12. Black elderberry (*Sambucus nigra*)	Polyphenols, anthocyanins, flavonols, tannins, and procyanidins	[126]
13. Cat’s claw (*Uncaria tomentosa*)	Proanthocyanidins, spiroxindole alkaloids, quinovic acid glycosides, Indole glycosides alkaloids, and tannins	[127]
14. Guduchi (*Tinospora cordifolia*)	Alkaloids, aliphatics, diterpenoid lactones, steroids, and glycosides	[128]
15. Holy basil (*Ocimum tenuiflorum*)	Phenols, flavonoids, polyphenols, eugenol, and methylchavicol	[129]
16. Milk thistle (*Silybum marianum*)	Flavonolignans, flavonoids, tocopherol, proteins, and sterols	[130]
17. Schisandra (*Schisandra chinensis*)	Flavonoids, phenolic acids, dibenzocyclooctadiene lignans, triterpenoids, and tannins	[131]
18. St. John’s wort (*Hypericum perforatum*)	Flavonoids, naphthodianthrones (hypericin), carbolic acids, phloroglucins (hyperforin), and xanthones	[132]
19. Triphala	Tannins, flavonoids, gallic acid, glucosides, chebulic acid, and quercetin	[133]
20. Wheatgrass (*Triticum aestivum*)	Flavonoids, chlorophyll, tannins, terpenoids, steroids, alkaloids, and glycosides	[134]
21. Myrrh	Steroids, terpenoids, flavonoids, lignans, and carbohydrates	[135]
22. Chamomile (*Matricaria chamomilla*)	Flavonoids, terpenoids, sesquiterpenes, phenolic acids, and coumarins	[136]
23. Ginkgo (*Ginkgo biloba*)	Flavonoids, ginkgolides, glycosides, terpenoids, and sesquiterpenes	[137]
24. Valerian (*Valeriana officinalis*)	Flavonoids, lignans, curcuminoids, tannins, phenolic acids, and quinones	[138]
25. Peppermint (*Mentha piperita*)	Menthol, flavonoids, phenolic acids, menthone, acetaldehyde, limonene, alkaloids, saponins, and glycosides	[139]
26. Saw palmetto (*Serenoa repens*)	Fatty acids (laurate, myristate, palmitate, linoleate), and phytosterols	[140]
27. Sage (*Salvia officinalis*)	Alkaloids, fatty acids, flavonoids, tannins, steroids, terpenoids, saponins, and coumarins	[141]
28. Aloe vera (*Aloe vera*)	Flavonoids, polysaccharides, saponins, vitamins, anthraquinones, fatty acids, salicylic acid, and lignins	[142]
29. Bilberry (*Vaccinium myrtillus*)	Anthocyanins, terpenoids, flavonoids, tannins, phenolic acids, and coumarins	[143]
30. Black cohosh (*Actaea racemosa*)	Terpenoids, phenolic acids, flavonoids, alkaloids, tannins, and aromatic acids	[144]
31. Feverfew (*Tanacetum parthenium*)	Sesquiterpene lactones, flavonoids, polyenes, and volatile oils (camphor, camphene)	[145]
32. Guggulu (*Commiphora mukul*)	Terpenoids, steroids, flavonoids, gluggultetrols, lignans, polysaccharides, and amino acids	[146]
33. Hawthorn (*Crataegus* spp.)	Phenolic acids, flavonoids, pyrocatechin, terpenoids, lignans, steroids, and organic acids (fumaric, tartaric)	[147]
34. Lavender (*Lavandula angustifolia*)	Phenolic acids, flavonoids, terpenoids (hydrocarbons, oxidated), Sesquiterpenes, amino acids, aldehydes, and coumarins (coumarin, herniarin)	[148]
35. Lemon balm (*Melissa officinalis*)	Volatile compounds (neral, geraniol, citronellal, geranial), flavonoids, phenolic acids, triterpenes, and tannins	[149]
36. Nettle (*Urtica dioica*)	Flavonoids, phenolic acids, amino acids, carotenoids, organic acids (Acetic acid, citric acid, formic acid), fatty acids, and tannins	[150]
37. Passion flower (*Passiflora incarnata*)	Alkaloids, flavonoids, phenolic compounds, cyanogenic glycosides, tannins, and steroids (β-Sitosterol)	[151]
38. Rhodiola (*Rhodiola rosea*)	Phenolic compounds, flavonoids, carotenoids, vitamin E, tannins, glycosides, organic acids, and salidroside	[152]
39. Slippery elm (*Ulmus rubra*)	Polysaccharides (D-galactose, L-rhamnose, D-galacturonic acid), phytosterols, and oleic and palmitic acids	[153]
40. Yarrow (*Achillea millefolium*)	Flavonoids, phenolic acids, terpenes (guaianolides, sesquiterpenes), phytosterols, organic acids, and fatty acids	[154]
41. Baikal skullcap (*Scutellaria baicalensis*)	Flavonoids (baicalein, baicalin, wogonin), phenylethanoid glycosides, polysaccharides, steroids, phenolic compounds, amine, and organic acids	[155]
42. Calendula (*Calendula officinalis*)	Terpenoids, steroids, flavonoids, triterpeneol esters, saponins, carotenes, carbohydrates, and tocopherols	[156]
43. Dandelion (*Taraxacum officinale*)	Sesquiterpene lactones, triterpenes, sterols, flavonoids, inulin, vitamins (A, C, E, K, B), and polyphenols (hydroxycinnamic acid)	[157]
44. Eleuthero (*Eleutherococcus senticosus*)	Saponins, phenylpropanoids, phenolic acids, polysaccharides, coumarins, lignans, and provitamins	[158]
45. Kava (*Piper methysticum*)	Flavonoids (flavokavains), kavalactones, and alkaloids	[159]
46. Marshmallow (*Althaea officinalis*)	Polysaccharides, flavonoids, phytosterols, tannins, coumarins, scopoletin, and amino acids	[160]
47. Red clover (*Trifolium pratense*)	Flavonoids, saponins, clovamides, phenolic acids, coumarins, and pterocarpans	[161]
48. Saw palmetto (*Serenoa repens*)	Fatty acids (free and esterified), triterpenes, flavonoids, carotenoids, tocopherols (Ve), and phytosterols	[162]
49. Wild yam (*Dioscorea villosa*)	Steroidal saponin (Diosgenin), allantoin, polysaccharides, and alkaloids	[163]

## Data Availability

Not applicable.

## Supplementary Materials

The following supporting information can be downloaded at: https://www.mdpi.com/article/10.3390/molecules28248045/s1, Figures S1–S10: Pro and anti-inflammatory cytokines affected by Echinacea, Astragalus, Maitake, Garlic, Shatavari, Ginseng, Ashwagandha, Licorice, Tulsi, and Ginger.
