# Supplementary material for: Medicinal Herbs: Promising Immunomodulators for the Treatment of Infectious Diseases
Source: Molecules. 2023 Dec 12;28(24):8045. doi: 10.3390/molecules28248045 (PMC10745476; doi:10.3390/molecules28248045)
Supplement: Supplementary file 1 [file molecules-28-08045-s001.zip › molecules-2641202-supplementary.pdf]

Supplementary files

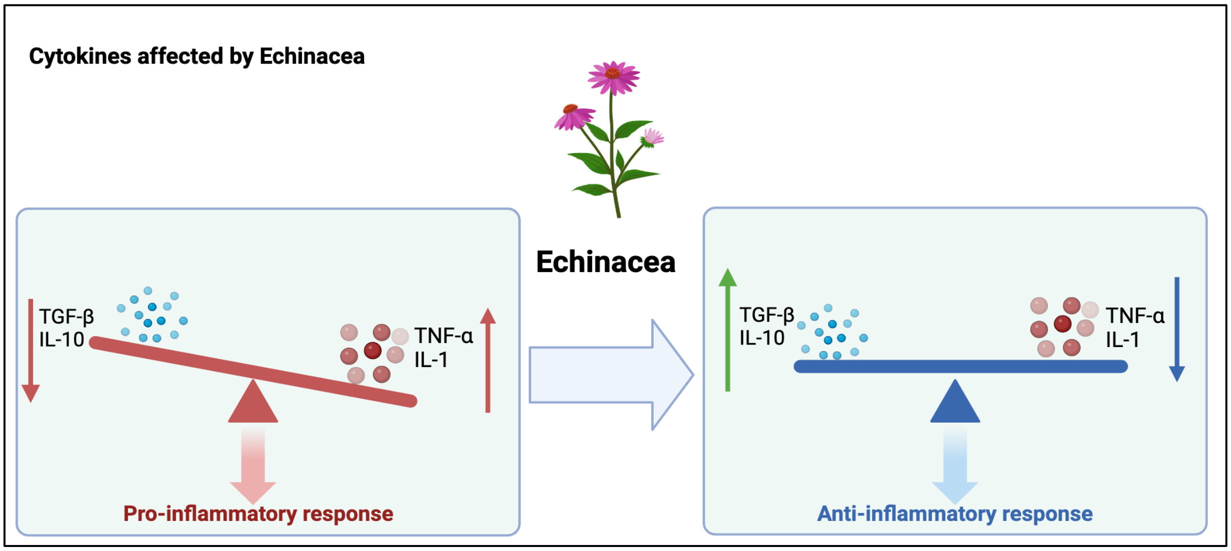

Figure S1. Pro and anti-inflammatory cytokines affected by Echinacea.

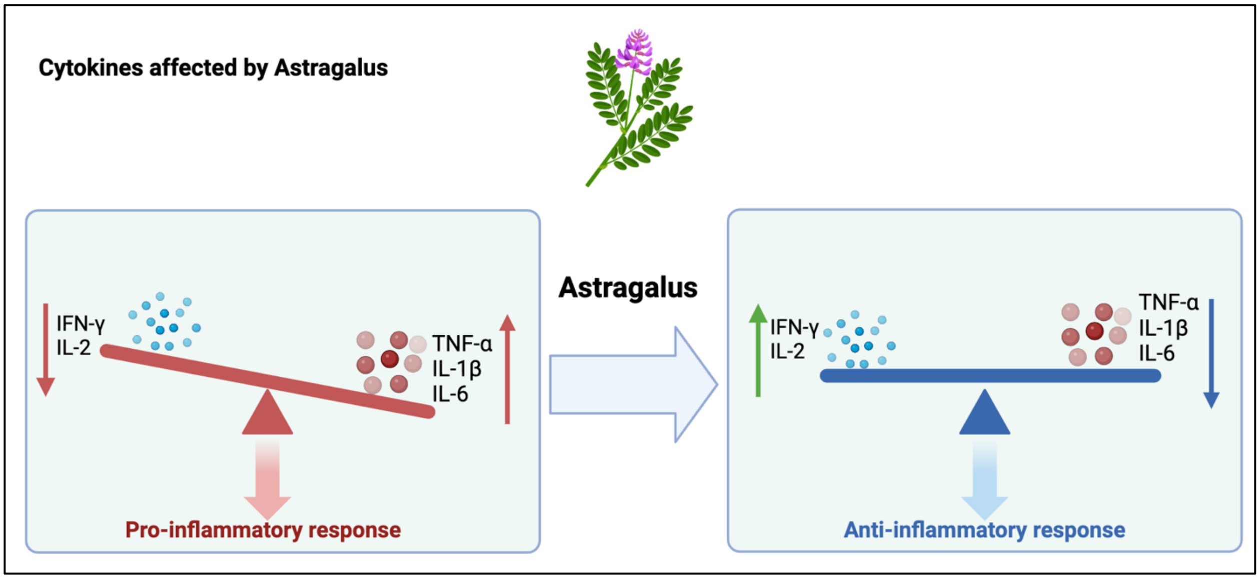

Figure S2. Pro and anti-inflammatory cytokines affected by Astragalus.

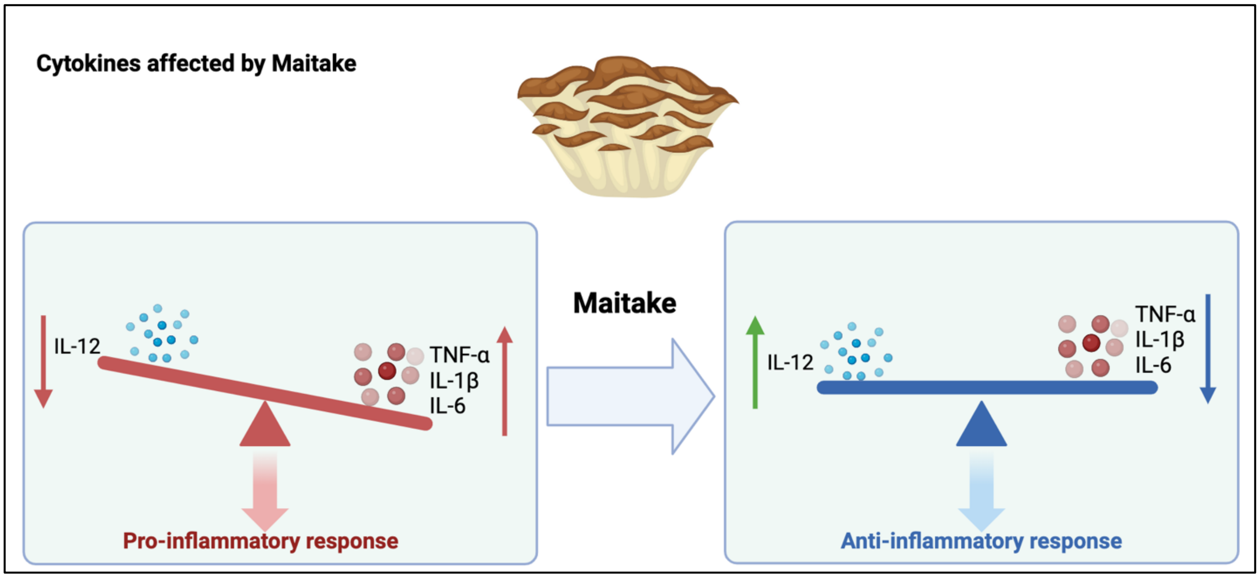

Figure S3. Pro and anti-inflammatory cytokines affected by Maitake.

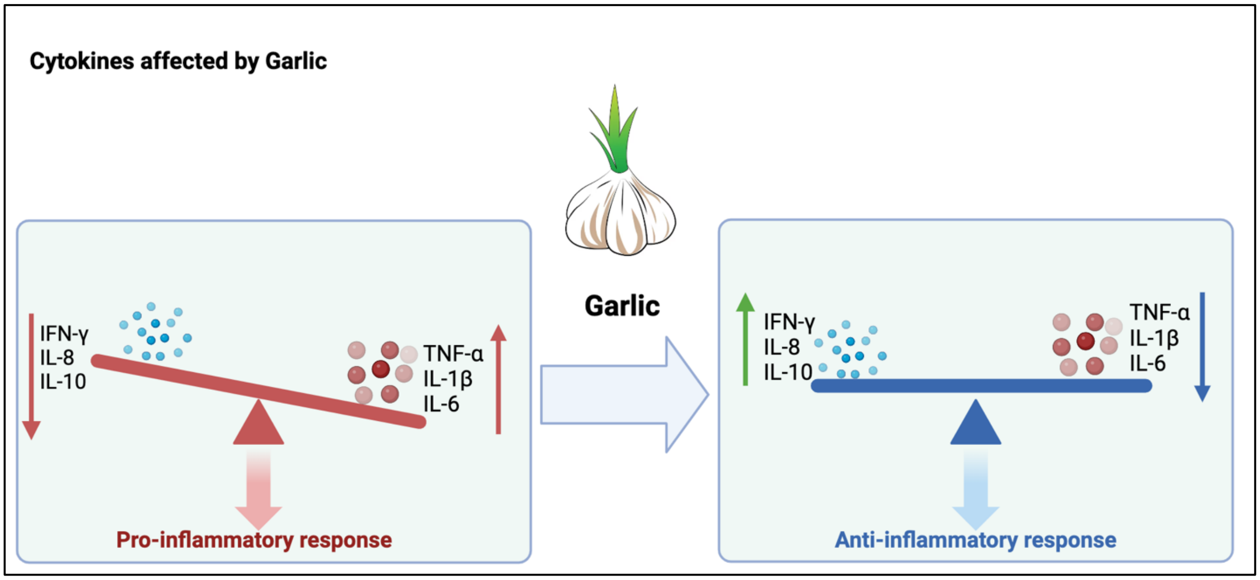

Figure S4. Pro and anti-inflammatory cytokines affected by Garlic.

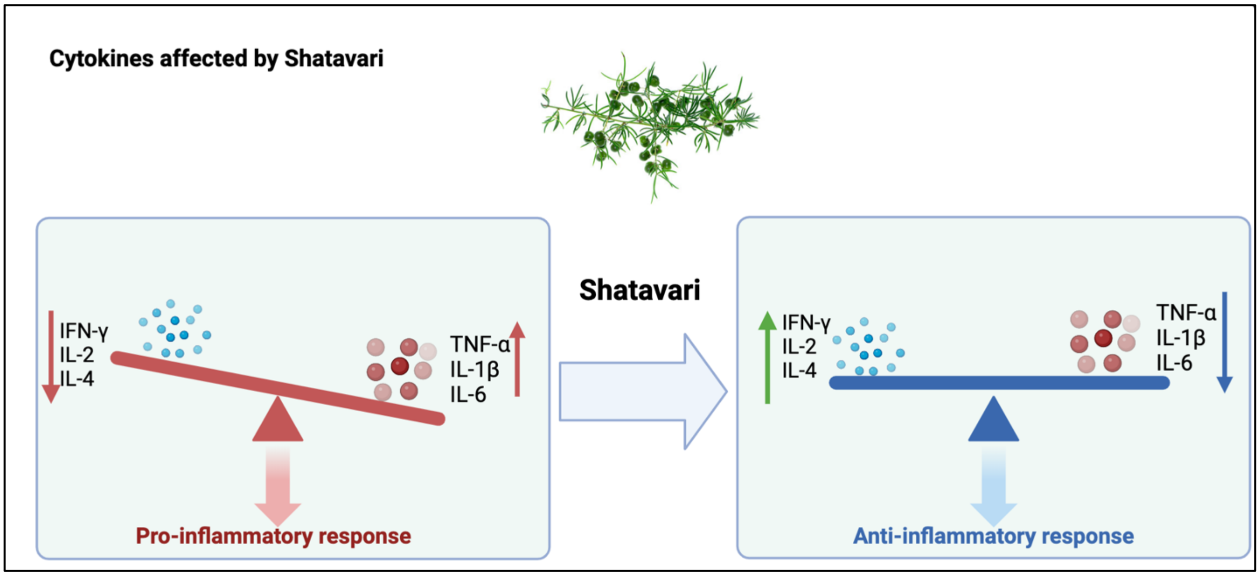

Figure S5. Pro and anti-inflammatory cytokines affected by Shatavari.

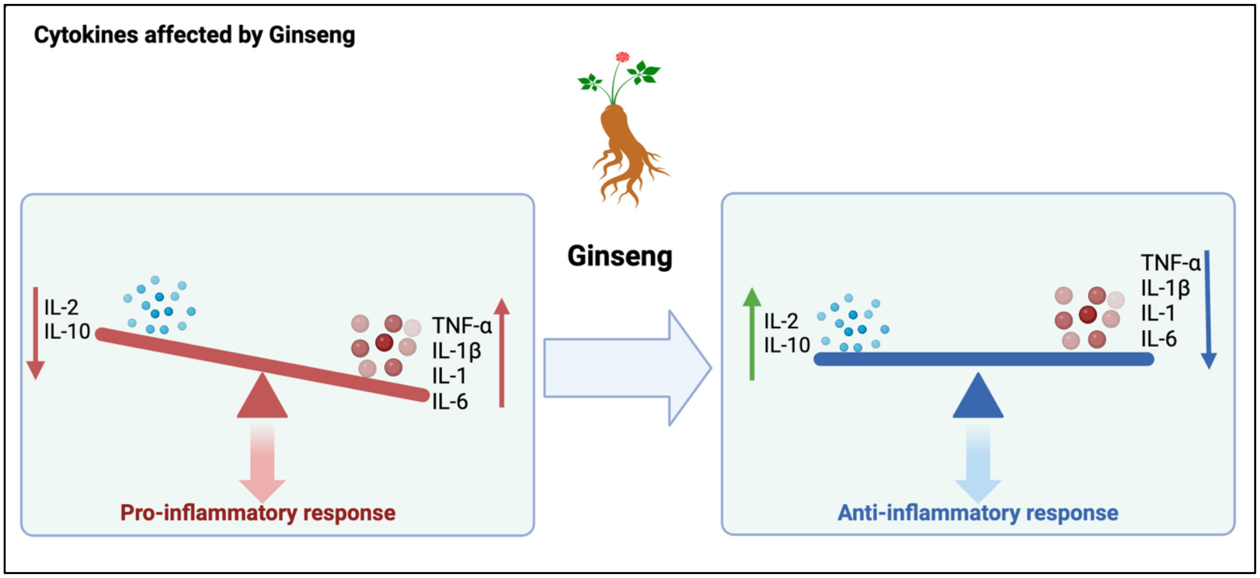

Figure S6. Pro and anti-inflammatory cytokines affected by Ginseng.

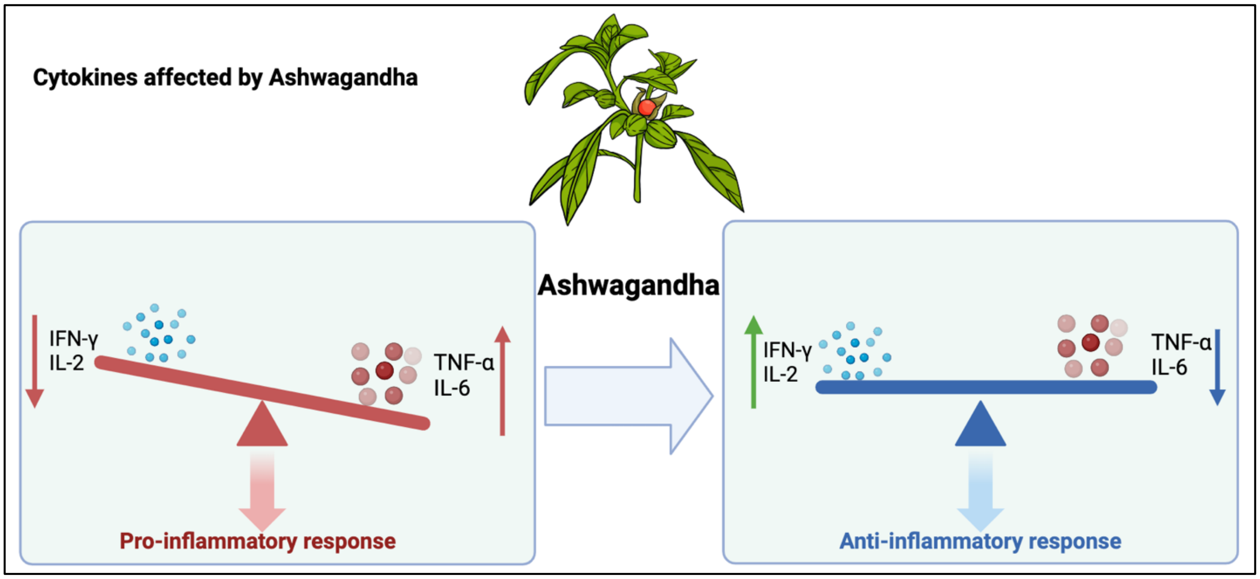

Figure S7. Pro and anti-inflammatory cytokines affected by Ashwagandha.

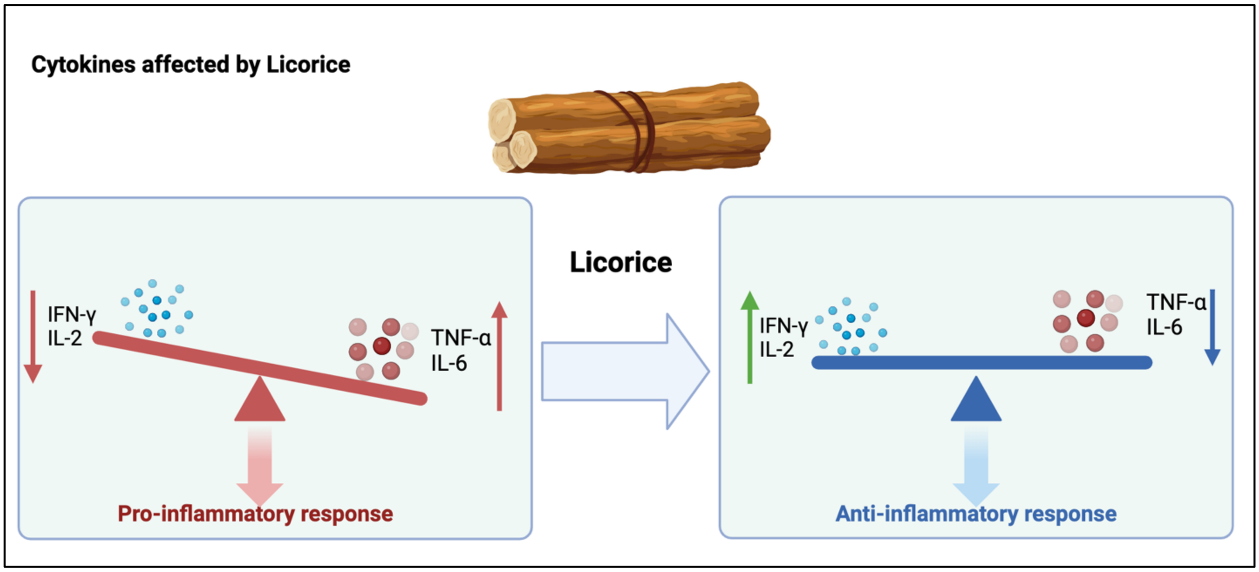

Figure S8. Pro and anti-inflammatory cytokines affected by Licorice.

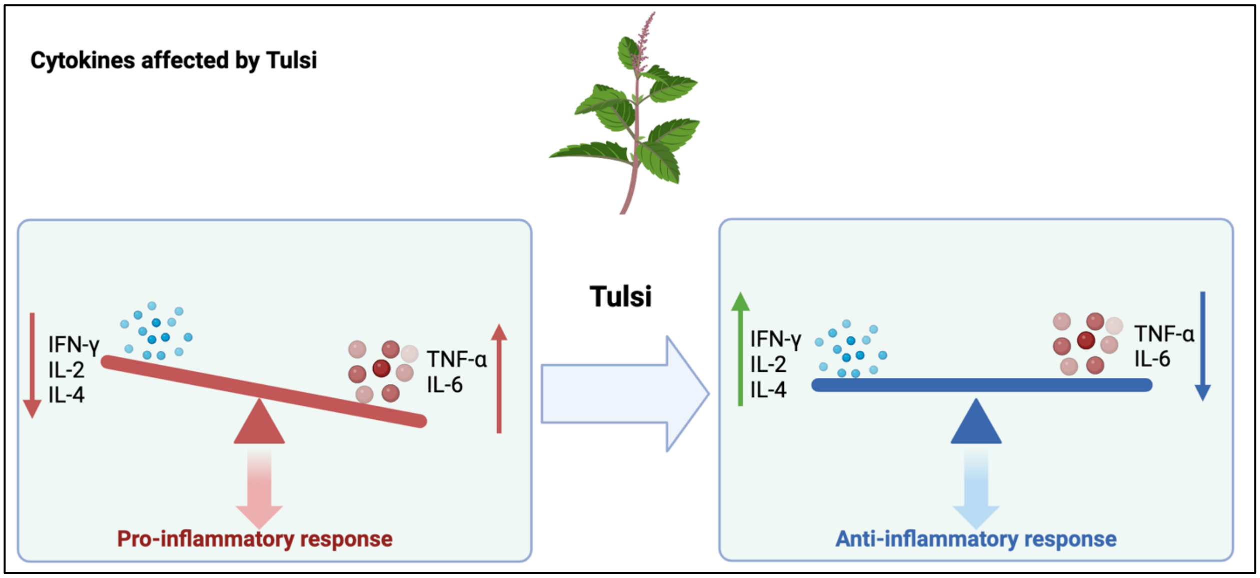

Figure S9. Pro and anti-inflammatory cytokines affected by Tulsi.

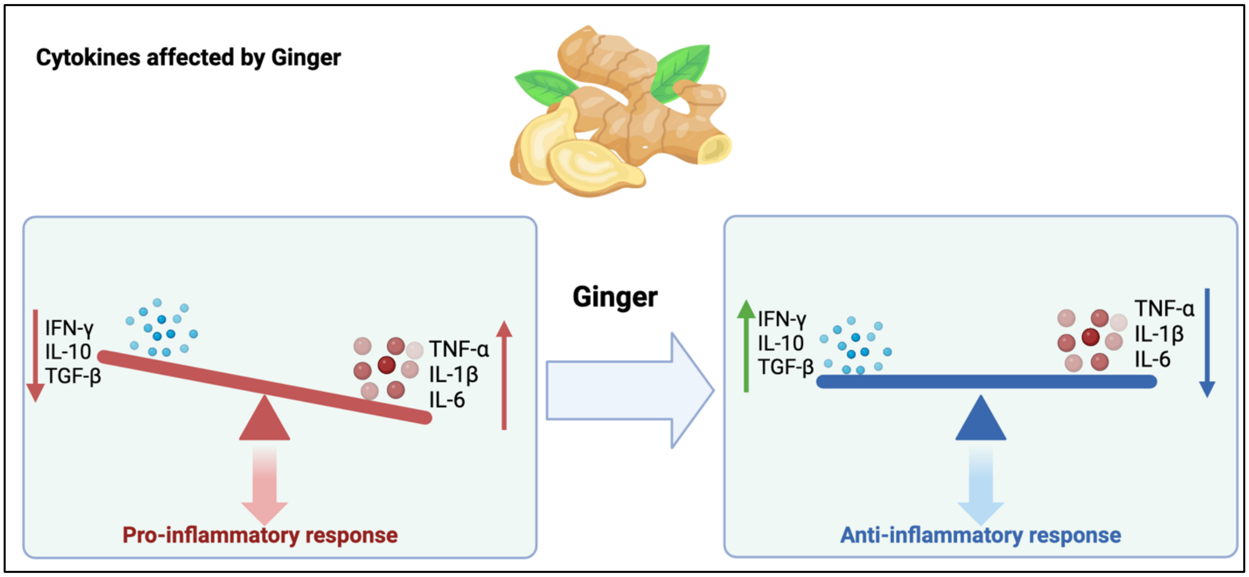

Figure S10. Pro and anti-inflammatory cytokines affected by Ginger.
